# Understanding the Effect of Adding Automated and Human Coaching to a Mobile Health Physical Activity App for Afghanistan and Iraq Veterans: Protocol for a Randomized Controlled Trial of the Stay Strong Intervention

**DOI:** 10.2196/12526

**Published:** 2019-01-29

**Authors:** Lorraine R Buis, Felicia A McCant, Jennifer M Gierisch, Lori A Bastian, Eugene Z Oddone, Caroline R Richardson, Hyungjin Myra Kim, Richard Evans, Gwendolyn Hooks, Reema Kadri, Courtney White-Clark, Laura J Damschroder

**Affiliations:** 1 Department of Family Medicine University of Michigan Ann Arbor, MI United States; 2 Durham Center of Innovation to Accelerate Discover and Practice Transformation Durham VA Health Care System Durham, NC United States; 3 Department of Population Health Sciences Duke University Medical Center Durham, NC United States; 4 Division of General Internal Medicine Department of Medicine Duke University Medical Center Durham, NC United States; 5 Division of General Internal Medicine Department of Medicine Yale University New Haven, CT United States; 6 Pain Research, Informatics, Multimorbidities, and Education Center VA Connecticut West Haven, CT United States; 7 VA Center for Clinical Management Research VA Ann Arbor Healthcare System Ann Arbor, MI United States

**Keywords:** exercise, veterans, cell phones, mobile phone, telemedicine

## Abstract

**Background:**

Although maintaining a healthy weight and physical conditioning are requirements of active military duty, many US veterans rapidly gain weight and lose conditioning when they separate from active-duty service. Mobile health (mHealth) interventions that incorporate wearables for activity monitoring have become common, but it is unclear how to optimize engagement over time. Personalized health coaching, either through tailored automated messaging or by individual health coaches, has the potential to increase the efficacy of mHealth programs. In an attempt to preserve conditioning and ward off weight gain, we developed *Stay Strong*, a mobile app that is tailored to veterans of recent conflicts and tracks physical activity monitored by Fitbit Charge 2 devices and weight measured on a Bluetooth-enabled scale.

**Objective:**

The goal of this study is to determine the effect of activity monitoring plus health coaching compared with activity monitoring alone.

**Methods:**

In this randomized controlled trial, with *Stay Strong*, a mobile app designed specifically for veterans, we plan to enroll 350 veterans to engage in an mHealth lifestyle intervention that combines the use of a wearable physical activity tracker and a Bluetooth-enabled weight scale. The *Stay Strong* app displays physical activity and weight data trends over time. Enrolled participants are randomized to receive the *Stay Strong* app (active comparator arm) or *Stay Strong* + *Coaching,* an enhanced version of the program that adds coaching features (automated tailored messaging with weekly physical activity goals and up to 3 telephone calls with a health coach—intervention arm) for 1 year. Our primary outcome is change in physical activity at 12 months, with weight, pain, patient activation, and depression serving as secondary outcome measures. All processes related to recruitment, eligibility screening, informed consent, Health Insurance Portability and Accountability Act authorization, baseline assessment, randomization, the bulk of intervention delivery, and outcome assessment will be accomplished via the internet or smartphone app.

**Results:**

The study recruitment began in September 2017, and data collection is expected to conclude in 2019. A total of 465 participants consented to participate and 357 (357/465, 77%) provided baseline levels of physical activity and were randomized to 1 of the 2 interventions.

**Conclusions:**

This novel randomized controlled trial will provide much-needed findings about whether the addition of telephone-based human coaching and other automated supportive-coaching features will improve physical activity compared with using a smartphone app linked to a wearable device alone.

**Trial Registration:**

ClinicalTrials.gov NCT02360293; https://clinicaltrials.gov/ct2/show/NCT02360293 (Archived by WebCite at http://www.webcitation.org/75KQeIFwh)

**International Registered Report Identifier (IRRID):**

DERR1-10.2196/12526

## Introduction

### Background and Significance

The type and intensity of physical activities performed by the veteran population changes considerably from active duty to postdeployment. This is often because of the relatively unstructured nature of postdeployment life, as well as service-connected illnesses or injuries [1]. Younger veterans involved in the Afghanistan and Iraq conflicts—Operation Enduring Freedom (OEF), Operation Iraqi Freedom (OIF), and Operation New Dawn (OND; OEF/OIF/OND)—may also have work-life balance issues related to childcare and elder care issues and additional civilian reintegration issues related to high physical and mental health burdens, which may present unique challenges to being more physically active (eg, chronic pain, mental illness, and substance abuse) [2,3]. The transition from being physically fit and active to being more sedentary can lead to rapid weight gain and an increased risk of adverse health outcomes including diabetes, heart disease, joint disorders, and some cancers. This rapid weight gain was documented in a prospective study by Litman et al (2013), where it was found that there is an increased rate of weight gain in veterans around the time of military discharge [4]. In a large cohort of OEF/OIF/OND veterans seen in the Veteran Health Administration (VHA) postdeployment, 65.8% of men and 46.7% of women were overweight or obese at their first visit [5]. Early intervention with individually tailored lifestyle programs targeting physical activity and prevention of weight gain has the potential to sustain the high level of fitness required for military service well past active-duty status and improve mental and physical health.

A potential strategy for increasing access to lifestyle modification programs for OEF/OIF/OND veterans and more technology savvy patients is through the use of mobile health (mHealth), which refers to the use of mobile computing, wearable sensors, and communication devices for the provision of health services and information [6]. Although mHealth apps are still emerging and evolving, there exists a solid foundation demonstrating that internet-mediated interventions [7], particularly when combined with the use of wearable physical activity monitors, tailored motivational messaging, and online coaching, can increase physical activity and have the potential to improve health outcomes [8-10]. Through leveraging the ubiquity of mobile devices, we can easily increase access to these types of interventions [6,11,12]. mHealth interventions have been built on findings from internet-mediated intervention trials and have likewise been shown to be effective in chronic disease self-management and promoting behavior change [13-22]; however, the evidence base for mHealth interventions is largely made up of small trials with short-term follow-up [6,18], and long-term engagement with mHealth programs is not always sustained [23,24]. A potential strategy for improving upon mHealth interventions is through the addition of health coaching, including telephone-based lifestyle coaching delivered by human coaches. Health coaching is a patient-centered, collaborative model grounded in theories of health behavior change, in which coaches work in partnership with patients to identify goals and action plans that maximize personal well-being and overall health. Many coaching interventions use techniques like motivational interviewing, goal setting, and problem-solving as key strategies. Across a wide variety of populations, health coaching has produced positive impacts on lifestyle modifications [25-27]. Adding automated coaching features, such as personalized messaging addressing barriers and motivators, as well as goal setting based on past outcomes gleaned from wearable devices, may further enhance the impact of health coaching [28].

### Specific Aims

We aim to evaluate the effect of adding automated (ie, automated tailored messaging and automated goal setting) and human telephone health coaching to *Stay Strong*, an mHealth app that seeks to improve physical activity in OEF/OIF/OND US veterans over 1 year. *Stay Strong* is a mobile app that is tailored to these veterans and tracks physical activity monitored by Fitbit Charge 2 devices and weight measured on a Bluetooth-enabled scale. Captured data are presented visually to participants to facilitate behavior change. We also aim to further assess the impact on the secondary outcomes of weight loss, depression, patient activation, and pain, as well as to test for potential moderating effects of individual characteristics (eg, demographics and familiarity with technology). We hypothesize that our enhanced version of the intervention *Stay Strong + Coaching* will result in greater improvement in physical activity (primary outcome) at 12 months compared with the base *Stay Strong* program.

## Methods

### Trial Design

This study is a randomized trial to test the effect of adding coaching (ie, automated tailored messaging, automated goal setting, and telephone calls with a health coach) to an mHealth intervention to improve and sustain levels of physical activity among a national sample of US veterans over 12 months. In this study, participants are randomized to receive either the base intervention (*Stay Strong*) or an enhanced intervention that includes coaching features (*Stay Strong + Coaching*). The decision to incorporate an active control group was guided by several considerations. First, there is sufficient evidence in the literature to suggest that this type of technology-mediated intervention for promoting increased physical activity is effective [8-10], even in a veteran population [29,30]. As such, we felt justified in not including a true usual-care control. Furthermore, physical activity tracking devices are highly ubiquitous in the general US adult population. On the one hand, the use of a wearable device by participants assigned to the control group would confound results. On the other hand, we did not believe it would be ethical to ask control-group participants to refrain from using one over the course of a 12-month intervention period. This study was reviewed and approved by the VHA Central institutional review board (IRB)–VHA IRB for multisite studies.

### Intervention

*Stay Strong* is a multicomponent mHealth physical activity intervention for iOS and Android platforms that supports physical activity and weight self-monitoring. Vibrent Health, a digital health company in Fairfax, VA, was engaged to develop the app, online portal, and server platforms necessary to support *Stay Strong* [31] (see Multimedia Appendix 1 for screenshots of the *Stay Strong* interface). The study team defined the functional system requirements to Vibrent, and then the team and Vibrent collaborated on the user-interface design. Feedback on the design was gathered from a convenience group of testers employed at the VA, several of whom were OEF/OIF/OND veterans, and the approach of utilizing a wearable activity tracker was endorsed by the target population in some of our previous work [32].

### Theoretical Foundations

**Figure 1:**
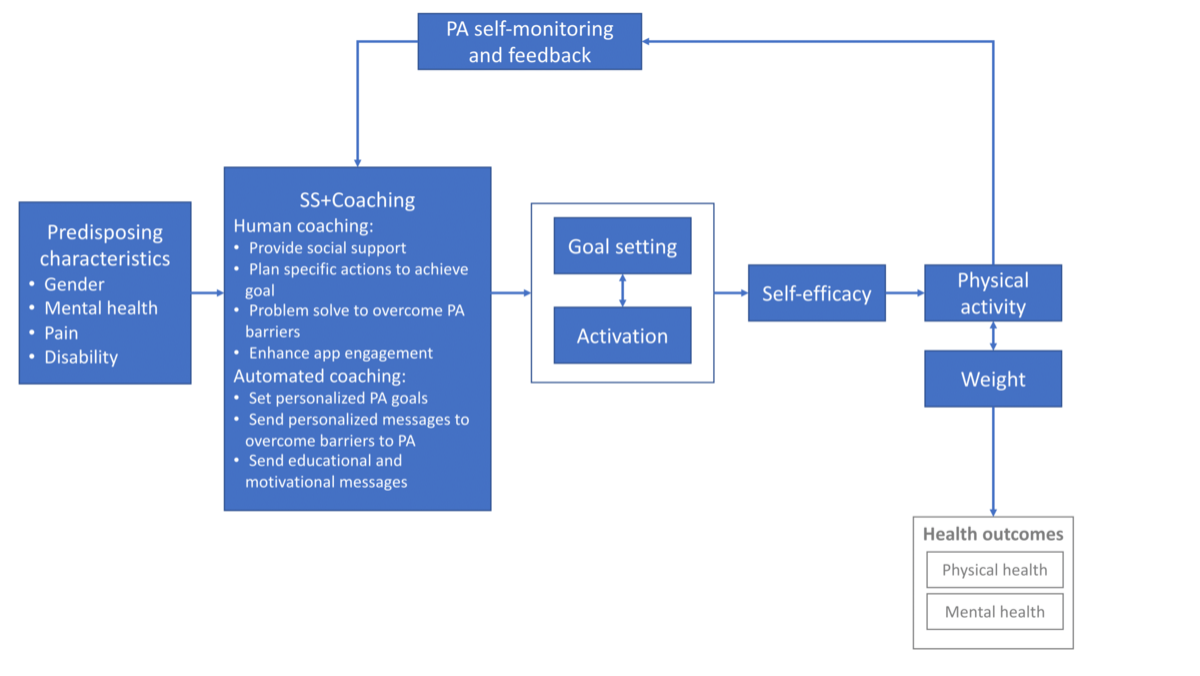
Theoretical framework. PA: physical activity; SS: *Stay Strong*.

Figure 1 shows the theoretical framework underlying the design of the intervention. The theoretical orientation of *Stay Strong* is informed by the information-motivation-behavioral skills (IMB) model and self-regulatory theory [33-36]. Both models have been successfully applied to physical activity and describe processes of behavior change mediated through goal attainment and skill mastery, and both models acknowledge the central role that self-efficacy plays in sustained behavior change [37-42]. The IMB hypothesizes that cognitive and behavioral skills are a prerequisite for any health behavior change like increasing physical activity. However, skills are the product of information that is relevant to health problems and personal and social support for activation to change behavior. Thus, information interacts with activation to enhance self-efficacy and build skill mastery to facilitate sustained behavior change. Informed by the pilot data and the IMB, *Stay Strong + Coaching* will boost the positive reinforcing relationship between self-monitoring and feedback with goal setting and activation through a coaching support component. Human coaches will enhance activation and facilitate goal setting by (1) providing social support, (2) helping to develop specific plans of action, (3) helping with problem solving to avoid or mitigate barriers to goal achievement, and (4) enhancing engagement with the *Stay Strong* app by helping to interpret the individuals’ physical activity data and using it to monitor progress toward their goals. Automated coaching will further strengthen these supports by (1) providing personalized, realistic physical activity goals, grounded in the objective physical activity monitoring provided through the Fitbit Charge 2 device, (2) sending personalized messages to help overcome barriers to physical activity, and (3) sending educational and motivational messages via the *Stay Strong* app.

The theory of self-regulation also informs the core components of *Stay Strong + Coaching*. In self-regulation, individuals participate in self-directed behaviors. These self-directed behaviors are hypothesized to be managed through a dynamic feedback loop in which individuals’ self-monitoring and feedback about their past behavior are integrated into their goals and activation to change future behaviors [43]. Thus, aligned with IMB, self-regulation involves both cognitive and behavioral processes to facilitate goal setting and attainment. A key strategy of *Stay Strong + Coaching* is to enhance activation and facilitate goal setting though enhanced self-monitoring. The Fitbit Charge 2 device will provide detailed minute-by-minute self-monitoring information through the objective measurement of physical activity. The self-monitoring and feedback loop will act on self-efficacy via 2 pathways: (1) influencing participants’ goal setting and activation to adhere to goals and (2) providing data for the human coaches and for automated coaching to customize motivational messages about goal attainment and to set appropriate, realistic future goals. Increased self-efficacy will mediate positive changes in physical activity, which will, in turn, lead to secondary outcomes of weight control and improved overall mental (eg, depression) and physical health (eg, pain) status. This theoretical grounding guided the choice of intervention components, which have each been classified in terms of an established taxonomy of behavior change techniques (BCTs) [44] (see Multimedia Appendix 2 for a complete list of BCTs by group assignment).

### Stay Strong and Stay Strong + Coaching Components

**Figure 2:**
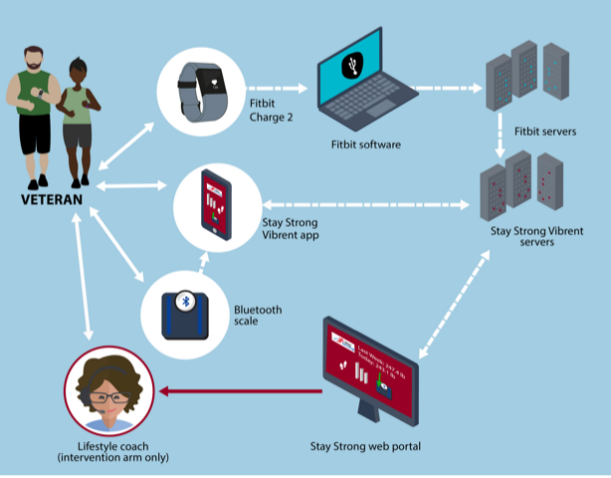
The *Stay Strong* intervention and data flow.

The *Stay Strong + Coaching* intervention comprises multiple components that support physical activity and weight self-monitoring. Figure 2 illustrates the components with data flows and Table 1 provides a complete list of intervention components by study arm.

**Table 1:** *Stay Strong* components by trial arm.

Component	SS^a^ Arm	SS + Coaching Arm	Intensity	Duration	Mode
Objective physical activity monitoring (Fitbit Charge 2 and data visualizations within SS)	✓	✓	Fitbit worn daily and data syncing at least once per week	1 year	Fitbit worn on wrist; Data visualizations available within the *SS* app
Weight self-monitoring (scale and weight data visualizations within SS)	✓	✓	Weight measured weekly with data syncing at least once per week	1 year	Data visualizations available within the *SS* app
Administrative message reminders (reminders for syncing, adverse event reporting, and data assessments)	✓	✓	1 message less than 230 characters	As needed over 1 year	Push notification on the smartphone
Automated personalized goal setting	—^b^	✓	Weekly, based on previous week’s and physical activity data	1 year	Abbreviated phone push notification plus message with image within the *SS* app
Automated messages: nonpersonalized	—	✓	1 message up to 225 characters, 3 per week	1 year	Abbreviated phone push notification plus a full message with visual image within the SS app
Automated messages: personalized, based on self-reported barriers	—	✓	1 message up to 225 characters, 3 per week	1 year	In-app and smartphone notification with image
Telephone-based lifestyle coaching	—	✓	Up to 30 min	3 calls in the first 9 weeks	Telephone

^a^SS: Stay Strong.

^b^Not applicable.

#### Stay Strong Components

##### Physical Activity Self-Monitoring

After enrollment, all the participants were provided with a Fitbit Charge 2 device, which is a wrist worn physical activity monitor that logs different objective measurements of physical activity, including *active minutes*, miles, steps, stairs, and heart rate zone. Data from the Fitbit device sync with the Fitbit platform and are then pulled into the *Stay Strong* app for viewing in table or chart view in 1- to 4-week increments. All the participants were encouraged to wear the Fitbit device during waking hours for the duration of the study period and upload device data at least weekly via the Fitbit Connect (Fitbit, Inc) software on their computer. Fitbit devices were chosen for their relatively low cost as well as their ubiquity in the consumer wearables marketplace, as well as in the research world [45], facilitating a path to implementation if the *Stay Strong* program is proven effective at improving physical activity in this population. Furthermore, previous work has demonstrated that although the Fitbit Charge HR (the previous version of the Charge 2 device) has its limitations compared with other wearable devices [46], it is suitable for research purposes as it provided an accurate measure of heart rate during walking and running activities [47].

##### Weight Self-Monitoring

After enrollment, all the participants were also provided a Bluetooth-enabled weight scale (A&D Deluxe Connected Weight Scale UC-352BLE). The data from the scale can sync with *Stay Strong* or be manually entered, and the data can be viewed in table or chart view in 1- to 4-week increments. All the participants were asked to use and sync their scale at least once per week during the study.

##### Automated Administrative Messaging Reminders

During the study period, all participants receive a variety of automated administrative messages, reminders to report any adverse events every 90 days, and reminders to complete 6- and 12-month survey assessments.

#### Stay Strong + Coaching Components

In addition to *Stay Strong* components, *Stay Strong + Coaching* participants receive the following components.

##### Automated Personalized Physical Activity Goals

*Stay Strong + Coaching* participants receive a new, automated and personalized daily physical activity goal, based on their previous physical activity, every Sunday morning at 9:00 am (based on the participants’ local time). New goals are calculated based on an average of the most recent 7 consecutive days where at least 5 of the 7 days have valid data (ie, at least 5 min of light activity). Physical activity goals specify the number of *active minutes* participants should seek to obtain every day. *Active minutes* is a proprietary measure that captures the number of minutes of continuous moderate-to-vigorous exercise when this level of activity is sustained for at least 10 min [48]. The minimum goal issued to participants is to average 10 *active minutes* per day. To minimize the risk associated with starting an exercise program and increase the likelihood of sustaining increased physical activity, automated goals guide participants to gradually increase their physical activity by no more than 5 *active minutes* per week. For safety reasons, no goal may exceed 60 *active minutes* per day, and physical activity goals set each week are never increased by more than 5 *active minutes* over the previous week’s goal. In the event participants have not synced physical activity data recently, automated reminders are sent. For participants who do not provide new data (either because of failure to sync data or nonadherence to wearing the Fitbit), the previous week’s goal will be reused. For participants who fail to meet their goal, their next goal may decrease or remain the same because goals are based on activity during the previous 7 days. Each week, goals are calculated based on data through Saturday, 11:59 pm local time. Participants are notified of their new goal through push notifications on their phone, and current goals are visually displayed within the *Stay Strong* app.

##### Automated Personalized Educational and Motivational Messaging

*Stay Strong + Coaching* participants receive 3 in-app and push notification messages per week. To keep messages fresh throughout the duration of the 12-month intervention and reduce the likelihood of participants ignoring messages sent at predictable times, messages are sent between 9:30 am and 5:30 pm (local time) Monday through Saturday at varying times. All messages are sent during the morning, afternoon, or evening, at a randomly selected time from the following options: morning=9:30 am, 10:30 am, 11:00 am, or 11:15 am; afternoon=1:00 pm, 1:45 pm, 2:40 pm, or 3:00 pm; evening=4:00 pm, 4:30 pm, 4:45 pm, or 5:15 pm. All messages are 225 characters or less including spaces, and they are designed to help the participants stay engaged in healthy lifestyle habits, as well as learn more about a variety of topics, including the following: (1) exercise, (2) healthy eating, (3) initiating behavior change, (4) pain, (5) inspirational quotes, (6) maintaining behavior change, (7) weight loss and weight management, (8) heart rate monitoring, (9) appropriate athletic gear, and (10) tips to overcoming self-identified barriers to physical activity. For messages pertaining to overcoming barriers to physical activity, the participants are asked to endorse up to 4 of a prespecified list of 11 barriers to increasing physical activity (lack of time, social influence, lack of energy, lack of willpower or motivation, fear of injury or pain, lack of resources, family obligations, weather conditions, depression, lack of accountability or external motivation, and disability) during the baseline and 6-month assessment. These barriers include the top 10 most common reasons why people are not more physically active, based on work by Sallis and Hovell (1990), Sallis et al (1992), and highlighted by the Centers for Disease Control and Prevention. In addition, we included 1 additional barrier related to disability [49,50].

##### Lifestyle Telephone Coaching Calls

*Stay Strong + Coaching* participants complete up to 3 phone calls with the *Stay Strong* coach within the first 9 weeks of the study. The *Stay Strong* coach has access to the participants’ survey responses and synced Fitbit data and works with participants to help them meet physical activity goals. The coach provides information to participants to enhance motivation through (1) assistance in developing goals and action plans to achieve Fitbit-derived physical activity goals, (2) assistance in problem solving barriers to achieving physical activity goals via tailored motivational support, and (3) guidance on features of the *Stay Strong* app, with an emphasis on interpreting Fitbit physical activity data outputs. In the event coaches are not able to reach participants by phone for coaching sessions, we attempt to contact participants via US mail.

### Participant Inclusion and Exclusion Criteria

To be eligible to participate in this study, participants had to be an OEF/OIF/OND veteran, identify a VHA medical center and VHA health care provider responsible for his or her care, be interested in starting a physical activity program in the next 30 days, have access to a computer with an internet connection and a working Universal Serial Bus (USB) port, have a smartphone running a compatible iOS or Android operating system, and be younger than the age of 65 years.

The decision to limit our recruitment to individuals under 65 years of age was because of higher risk of cardiac issues in individuals over the age of 65 years. The individuals were excluded if they reported that a health care provider had told them that it was currently unsafe to exercise in an unsupervised or unmonitored setting, they had a history of eating disorders or a body mass index less than 20, they were not competent to consent for themselves to a research study, or have worn a physical activity sensor within the last 30 days.

### Recruitment Procedure

#### Sample Size Calculation

Our sample size estimate is based on the primary hypothesis: OEF/OIF/OND veterans randomized to the *Stay Strong + Coaching* intervention arm will have greater mean daily minutes of *active minutes* (moderate-to-vigorous physical activity) at 12 months than veterans randomized to the *Stay Strong* arm. A 10-min differential improvement at 12 months was set as a minimal clinically important difference. This represents the differential between the intervention and comparison arms at 12 months; therefore, the power and sample size considerations apply even if the comparison arm also improves over the study period. The mean daily minutes at 12 months will be obtained based on an analytic model using weekly averages of activity per day over the 12-month study period with up to 52 weeks of data. Sample size calculations are based on analysis of covariance methods as described in Borm’s paper, and are thus conservative [51]. We used data from the Lifestyle Education for Activity and Nutrition study [52] and our earlier unpublished feasibility study to estimate quantities needed for the sample size calculation. Our sample size calculations were based on unpublished data from a pilot study led by members of our team using a Body Media Fit device [32]. Although these calculations are based on a different device, we expect the SD of *active minutes* (our primary outcome) or the clinically meaningful and detectable difference, to be similar for interventions using the Body Media Fit versus the Fitbit Charge 2 device. On the basis of this unpublished pilot data, we anticipated a baseline mean of 53 min, a SD of 28 min in both treatment groups, .46 correlation between baseline and 12 months, and 25% attrition by 12 months based on previous studies. The study is designed to randomize 350 patients (175 per group) to detect with a .05 level 2-sided test, with a 10-min difference in improvement at 12 months with 90% power.

#### Participants

To recruit participants, we used the OEF/OIF/OND roster from the VHA Corporate Data Warehouse to identify candidate participants who had been seen in VHA primary care in the past year. Using scrambled social security numbers from the OEF/OIF/OND roster, we retrieved current mailing addresses through the VHA Informatics and Computing Infrastructure in batches of 2000. The pool of candidate participants was selected to proportionally represent each of the eligible VHA sites across the continental United States and Puerto Rico. It should be noted that the majority of letters intended for potential participants from Puerto Rico were returned as undeliverable or were intentionally not sent by the study team because of Hurricane Maria in September 2017. We oversampled women to achieve a targeted proportion of 20%. The enrolled individuals were randomly assigned to 1 of 2 groups in a 1:1 ratio: (1) the active control group (*Stay Strong*) or (2) the intervention group (*Stay Strong + Coaching*). The high targeted number of consented individuals (n=750) was because of anticipation that many individuals were likely to drop out because of the number of technical steps that must be completed before randomization including downloading and installing the *Stay Strong* app on their smartphone, pairing their Fitbit device to the app and syncing sufficient valid activity data in a 7-day period to capture baseline activity.

### Study Recruitment, Enrollment, Randomization, and Retention

Recruitment packets containing an invitation letter and a brief study overview sheet were sent in batches of approximately 200. First, the candidate participants were provided an individually assigned program code in their invitation letter and directed to a website. Second, the individuals completed a set of screening questions to assess study eligibility. If they were eligible, they were then directed to the online consent and Health Insurance Portability and Accountability Act (HIPAA) authorization processes and the baseline survey. Next, participants were instructed to install and register the *Stay Strong* app through Google Play (Android smartphone users) or Apple (iPhone users) stores. When the participants successfully installed the *Stay Strong* app on their smartphone, the study team was automatically notified via an online portal. At this point, the participants were considered to be fully enrolled if they completed all of these steps. All the participants were reminded to follow-up with their health care provider as needed throughout the study.

The participants who were fully enrolled were then shipped a package that included a welcome letter, devices (Fitbit Charge 2 & Bluetooth weight scale), a Fitbit USB dongle for syncing their Fitbit device, instructions, a frequently asked questions document, a written copy of the Study Information Sheet, and HIPAA authorization. The participants were required to authorize the *Stay Strong* app to sync and access data from Fitbit. When they were fully configured, the participants were instructed to use their devices for up to 2 weeks and to sync their data via the Fitbit Connect software, at least weekly. When 7 consecutive days having at least 5 valid days of data were synced, the individual was randomized to *Stay Strong* or *Stay Strong + Coaching*. A day’s worth of data were deemed valid if at least 5 lightly *active minutes* had been recorded. The individuals were instructed to sync data via the Fitbit Connect software only via a Bluetooth-enabled dongle, which required access to a computer using a USB port. This was necessary to comply with VHA data security and confidentiality standards.

If the participant had not met the minimum criteria to be randomized after 2 weeks, the study staff would attempt to call the individual to solve any technical issues that had arisen. A total of 3 attempts were made to contact the individual and receive the physical activity data. Our intent was to randomize the participants who successfully completed all the steps and met all the criteria. Randomization was stratified by gender, physical activity level (high vs low), and smart phone operating system (android vs iOS) into the 2 groups in a 1:1 ratio, with a target of randomizing 175 participants per arm. All the study staff were blinded to the randomization list, which was created by the study statistician using Stata 14.1 (StataCorp LLC) with random block sizes and uploaded into the study tracking database by the programmer. After an individual met the requirements for randomization, a staff member requested the arm assignment using an automated system within the study tracking database. After the assignment was made, the staff updated the mobile app to comply with the assigned arm. The study staff did not have any in-person interactions with the participants during the course of the study and had specific telephone contact protocols to protect against potential bias.

All the study participants will be provided with a US $25 Amazon gift card at 6- and 12-months for completing the study follow-up surveys. The participants will be permitted to keep the Fitbit device and scale that were issued as a part of this study.

### Data Collection

Online survey data for this study are collected at baseline, as a part of the enrollment process, and again at 6- and 12-months postrandomization. In the event participants fail to complete the online assessments and are unreachable via phone, we will attempt to contact the participants via US mail. After completing the 12-month data collection survey, as well as a final Fitbit device upload, all participants’ study-issued Fitbit accounts are deleted and the participants are able to setup their own personal account at their discretion. The *Stay Strong* app for both groups will remain active for an extra 30 days to ensure that sufficient endpoint physical activity and weight data are synced.

### Measures

Within this study, we will collect a variety of outcome measures, which are summarized in Table 2.

**Table 2:** Outline of measures and data collection points for the *Stay Strong* trial.

Measure	Source	Timepoints assessed	Method of measurement
Demographic measures	Online survey	Baseline	Self-report
Active minutes [48]	Fitbit	Continuous	Wearable sensor
Body weight	Bluetooth scale	Daily or as assessed by user	Synced data from scale or manually entered
Patient activation measure	Online survey	Baseline, 6 and 12 months	Self-report
Motivation to change health behaviors [53]	Online survey	Baseline, 6 and 12 months	Self-report
Social support general (ISEL-12^a^) [54]	Online survey	Baseline, 6 and 12 months	Self-report
Physical activity questions including exercise vital signs [55]	Online survey	Baseline, 6 and 12 months	Self-report
Self-regulation exercise and diet [56]	Online survey	Baseline, 6 and 12 months	Self-report
Self-efficacy and controllability [57]	Online survey	Baseline, 6 and 12 months	Self-report
Social support for diet and exercise [58]	Online survey	Baseline, 6 and 12 months	Self-report
Diet—starting the conversation [59]	Online survey	Baseline, 6 and 12 months	Self-report
Physical activity [60]	Online survey	Baseline, 6 and 12 months	Self-report
Self-perception of weight [61]	Online survey	Baseline, 6 and 12 months	Self-report
General health via SF-12^b^ [62]	Online survey	Baseline, 6 and 12 months	Self-report
Depressive symptoms (PHQ-8^c^) [63]	Online survey	Baseline, 6 and 12 months	Self-report
Pain, pain intensity, and pain interference with daily activities [64-67]	Online survey	Baseline, 6 and 12 months	Self-report
Sleep (MOS-6^d^) [68]	Online survey	Baseline, 6 and 12 months	Self-report
Smoking and alcohol use [69,70]	Online survey	Baseline, 6 and 12 months	Self-report
Technology use, comfort, and acceptance [71,72]	Online survey	Baseline, 6 and 12 months	Self-report
Physical Activity devices	Online survey	Baseline, 6 and 12 months	Self-report
Barriers to physical activity [73,74]	App-based survey for Intervention only	Baseline, 6 and 12 months	Self-report

^a^ISEL-12: 12-item Interpersonal Support Evaluation List. * *

^b^SF-12: 12-Item Short-Form Health Survey.

^c^PHQ-8: 8-item Patient Health Questionnaire.

^d^MOS-6: 6-item Sleep Scale from the Medical Outcomes Study.

#### Physical Activity

Our primary outcome of interest is physical activity from baseline to 1-year postrandomization, as measured by average daily *active minutes* on the Fitbit device. Specifically, we will compare with groups the *daily active minutes* at 12 months (primary endpoint) and change in daily *active minutes* from baseline to 12 months (slope) based on data measured weekly, averaged over a 1-week period based on availability of at least 5 days of valid data (ie, requiring at least 5 min of light activity within each day). As a secondary measure of physical activity, we will also compare average step counts from baseline to 12 months.

#### Weight Loss

Weights are objectively assessed using the study-issued Bluetooth-enabled scale (A&D scale) and can be automatically synced with *Stay Strong*. Users also have the option of manually entering their weight within the app. Weekly average weights will be calculated similarly to weekly *active minutes*.

#### Depression

The presence and severity of depression will be measured by the 8-item Patient Health Questionnaire (PHQ-8) [63]. The PHQ-8 is the same as the widely used 9-item PHQ (PHQ-9), with the removal of 1 item focused on suicidality. The PHQ-9 is a widely used tool for screening, diagnosing, monitoring, and measuring the severity of depression, and it has been shown to be a reliable and valid measure [75].

#### Patient Activation

Change in patient activation will be assessed using the Patient Activation Measure (PAM) developed by Hibbard et al [76]. The PAM is a 13-item measure that assesses knowledge, skills, beliefs, and confidence for managing an individual’s own health. PAM scores have demonstrated high construct validity, and they are highly correlated with individual’s engagement in healthier lifestyle behaviors [77].

#### Pain

Pain is often a barrier to physical activity, but higher levels of physical activity can help reduce chronic pain [78]. We will utilize the VHA Standard Pain Frequency and Intensity scale to assess pain.

### Statistical Analysis Plan

The primary analytic cohort will be intent-to-treat, with the proposed primary and secondary analyses focusing on the effect of *Stay Strong + Coaching* compared with *Stay Strong* alone. The 1 exception is with women who self-report pregnancy at any of the 3 assessment times (baseline, 6-month, and 12-month); they will not be included in the analyses for the primary outcome of physical activity or for weight loss.

Descriptive statistics, including graphical displays, will be used to summarize all study variables overall and by arm. Evidence of between-arm imbalance in baseline characteristics that are potentially related to change in physical activity level (eg, baseline weight and full-time work status) will be noted and sensitivity analyses adjusting for these baseline characteristics will be considered to ensure that an observed intervention effect is not because of this baseline imbalance. Summary statistics will also be reported for primary and all secondary outcomes by arm, including unadjusted changes in daily *active minutes* from baseline to 12 months as means and 95% CIs.

Between-group comparison of *active minutes* will be examined using all longitudinally assessed weekly averages of *active minutes*, including the baseline *active minutes*, based on the longitudinal data mixed-effects model (75) with time (weeks since randomization), treatment group, and interaction of time by treatment group as primary predictors, participants and slopes as random effects, and autoregressive correlation within the person. The model will be adjusted for stratification factors of sex, operating system, and baseline activity level (high vs low). Time may be parameterized appropriately if a nonlinear slope over time is suggested in graphical examination of the outcome measures over time. On the basis of the model, predicted *active minutes* at 12 months will be compared with 2 treatment groups, and a test of significant slope of the interaction term will also be used to test if the change from baseline in *active minutes* differ between treatment groups. Weights will be analyzed similarly. Although assessments will be done only 3 times, the secondary outcomes of depression and pain will be analyzed similarly as for the analyses for *active minutes*. To test for moderation of the coaching effect by gender, the model specified above will be expanded to include the gender by *Stay Strong + Coaching* interaction. We also plan to conduct analyses to examine patterns of intervention utilization and dose-response relationships. All the statistical analysis will be completed by the study team, independent of the funder and vendors involved in the study.

### Adverse Event Monitoring

The participants are instructed to report any changes in their medical condition to their primary health care provider first, followed by a report to the study hotline. Moreover, all participants receive automated in-app reminders every 90 days to notify the study staff of health changes. Any reported adverse event will be followed up by the study staff for additional information and will be classified as serious or not serious, related or unrelated, and anticipated or unanticipated, as well as severity, by the study staff and study physicians. Any adverse event that is categorized as serious is subject to IRB required reporting, and medical suspension and reclearance processes will be initiated as required.

## Results

**Figure 3:**
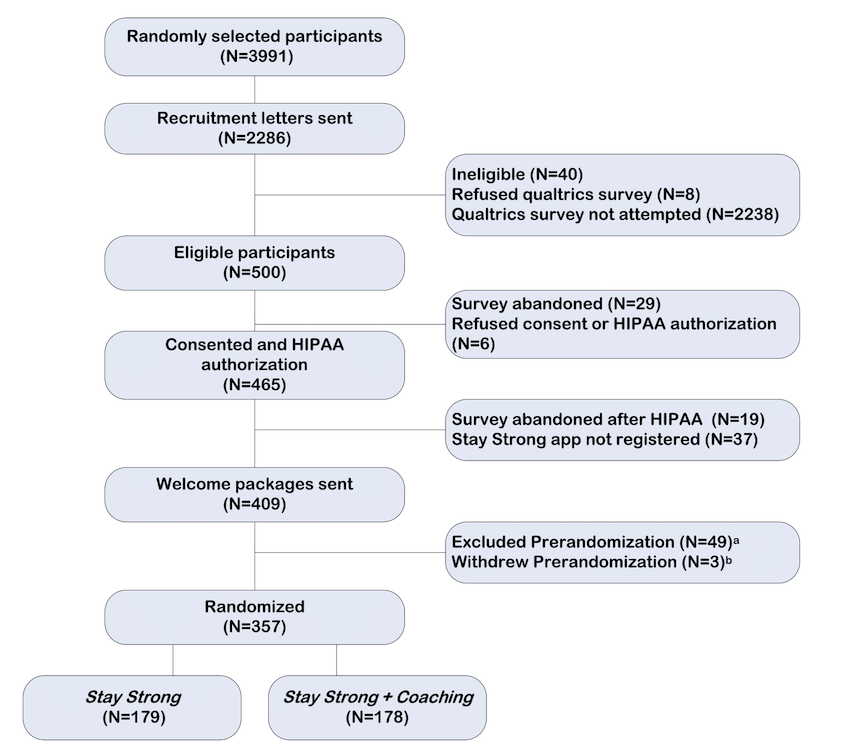
Participant flow through recruitment, enrollment, and randomization. HIPAA: Health Insurance Portability and Accountability Act. Note: a: Unable to sync Fitbit (N=12), did not pair with study account (N=12), unable to setup device due to secure environment (N=1), unable to contact (N=9), unable to setup Fitbit with *Stay Strong* app (N=10), unable to comply or follow study procedures (N=5); b: Changed mind about participating (N=3).

The participant recruitment began in September 2017 and concluded in May 2018. Figure 3 shows the flow of patients from initial contact to randomization. In total, 2286 invitation letters were sent, and 540 potential participants completed the eligibility screening, 23.62% (540/2286) response rate. Of those who completed the eligibility screening, 7.4% (40/540) were screened as ineligible based on their survey responses. Of the remaining 500 eligible potential participants, 81.8% (409/500) of the individuals consented to participate in the study, provided HIPAA authorization, and registered the *Stay Strong* app by installing it on their smartphone. Of those, 87.3% (357/409) provided valid baseline physical activity levels and were randomized to *Stay Strong* (n=179) or *Stay Strong + Coaching* trial arms (n=178). Currently, all the participants have completed at least 9 weeks of the trial, and all coaching calls are complete. Data collection is expected to conclude in 2019.

## Discussion

### Trial Implications

This study marks a significant contribution to the mHealth literature. Despite the increasing ubiquity of wearable physical activity tracking devices among consumers, recent reviews identified the need for randomized trials with a sufficiently large sample size and longer duration (longer than 12 weeks) interventions [6,28]. Moreover, the lack of fully described theoretical frameworks that underlie mHealth-mediated behavior change interventions has been noted [79,80]. *Stay Strong* is a fully powered trial with an active comparator, which follows participants for 12 months [28]. It features a theoretically driven behavior change intervention. Furthermore, most trials using wearables include samples with a minority of men, whereas participants in this trial are predominately male (267/357, 74.8%), which is an especially important contribution to the literature. Also, we seek to assess if coaching provides additional benefits via deeper engagement with wearable activity monitors compared with activity monitoring alone, a unique contribution to the literature.

This study is also significant in its reliance on a national sample of OEF/OIF/OND veterans who were consented and enrolled online with no in-person assessments or interactions. On the whole, OEF/OIF/OND veterans are younger than other veteran cohorts. Publicly available data show that post-9/11 veterans have a median age of 35 years; thus, many are *digital natives* born after 1980, having grown up with computers and other forms of information technology [81]. Recent estimates indicate that 98% of American adults aged 30-49 years have a cell phone, and 89% have a smartphone, suggesting that our smartphone-based intervention is well suited to our targeted population, important for increased adoption and uptake [82].

The response to mailed letters was much higher than anticipated based on our team’s past experiences with recruiting for internet-mediated interventions. This has been especially striking, given that it can be challenging to engage veterans in lifestyle interventions as demonstrated by our work and the work of others. The average age of participation in VHA’s weight loss program (also known as MOVE!) is 57 years [83], compared with 37 years for *Stay Strong* participants. Thus, mHealth interventions have significant potential for engaging this cohort of veterans to improve lifestyle behaviors like physical activity. The *Stay Strong* trial is the first national trial of an mHealth intervention in the VHA aimed at increasing physical activity.

### Limitations

This study has limitations. Our primary outcome measure of physical activity is focused on *active minutes*, as measured by the Fitbit device. As previously mentioned, *active minutes* is a proprietary measure that is not precisely defined, and any potential changes to how *active minutes* are calculated by Fitbit are out of our control. Although this is a limitation to our approach, there is also strength in the fact that our use of this measure allows us to ensure that this nationally-conducted randomized trial can be conducted entirely without bringing participants into a clinic or research setting for data collection. This pragmatic nature opens the door to a more nationally representative sample and reduces the ultimate burden placed on research participants. In this vein, another limitation to our approach is our reliance on self-reported data for some of our collected measures. We have taken steps to gather objectively measured data whenever possible (via study issued devices). Another limitation of our approach centers on the fact that not all coaching features persist after the first 2-3 months of the intervention. Although our automated features continue through the duration of the program, contact with human coaches is focused on in the first several weeks. Future work should seek to determine whether prolonged human-coach contact has an effect on participant engagement and participant outcomes. We also have a methodological limitation because we seek to determine the effectiveness of adding coaching features to the *Stay Strong* intervention and we are predominately focused on quantitative measures. Although we understand the richness that a qualitative or mixed-methods approach would add to this study, budgetary constraints preclude additional measures or qualitative data collection. Additionally, our approach is limited by the fact that participants are instructed to sync their data using a dongle attached to a laptop or desktop computer rather than syncing directly using the Fitbit app on their smartphone. This was necessary in our setting to help ensure privacy of personal data and comply with VA regulations. This decision will likely affect our user engagement and compliance with syncing protocols because of the increased burden in using this approach. The reliance on laptop or desktop syncing reduces generalizability and potentially disproportionally excludes lower-income individuals, who are more likely to rely on cell phones for internet connectivity and are less likely to own desktops or laptops. Lastly, though we intended to block-randomize participants by gender, physical activity level, and smartphone operating system into the 2 groups in a 1:1 ratio, the first 283 participants were randomized without blocks because of a programming error. However, the last 74 participants were block randomized. This led to unequal distributions in a few of the *cells*. However, analyses showed there were no differences in baseline characteristics.

### Conclusions

This randomized control trial will provide much-needed findings about whether the addition of telephone-based plus automated coaching and personalized goal setting will improve levels of physical activity compared with using a wearable device and smartphone app alone. This year-long trial will also provide insights on feasibility and acceptability of interventions like *Stay Strong* among OEF/OIF/OND veterans.

## Appendix

Multimedia Appendix 1 *Stay Strong + Coaching* screenshots.

Multimedia Appendix 2 Behavior change techniques (BCTs) by *Stay Strong* intervention component.

## Abbreviations

BCT: behavior change technique
HIPAA: Health Insurance Portability and Accountability Act
IMB: information-motivation-behavioral skills
IRB: institutional review board
mHealth: mobile health
OEF: Operation Enduring Freedom
OIF: Operation Iraqi Freedom
OND: Operation New Dawn
PAM: Patient Activation Measure
PHQ-8: 8-item Patient Health Questionnaire
PHQ-9: 9-item Patient Health Questionnaire
USB: Universal Serial Bus
VHA: Veterans Health Administration
