# Supplementary material for: Understanding the Effect of Adding Automated and Human Coaching to a Mobile Health Physical Activity App for Afghanistan and Iraq Veterans: Protocol for a Randomized Controlled Trial of the Stay Strong Intervention
Source: JMIR Res Protoc. 2019 Jan 29;8(1):e12526. doi: 10.2196/12526 (PMC6371069; doi:10.2196/12526)
Supplement: Multimedia Appendix 1 [file resprot_v8i1e12526_app1.pdf]

Multimedia Appendix 1: *Stay Strong* + *Coaching* Screenshots

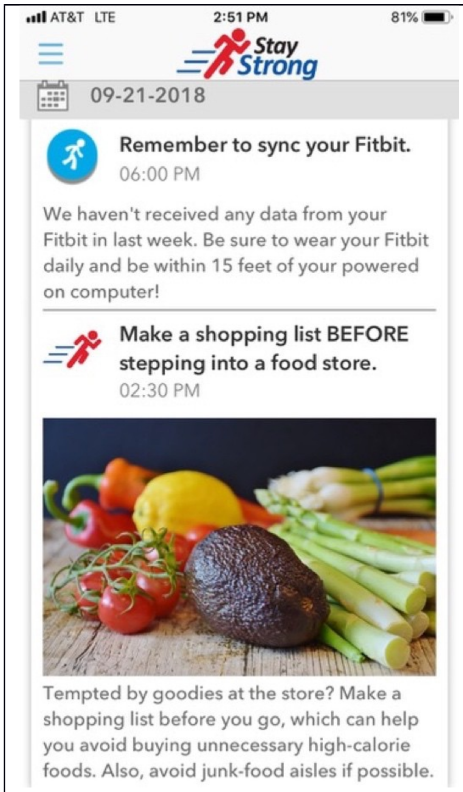

Wall with sync reminder and educational message

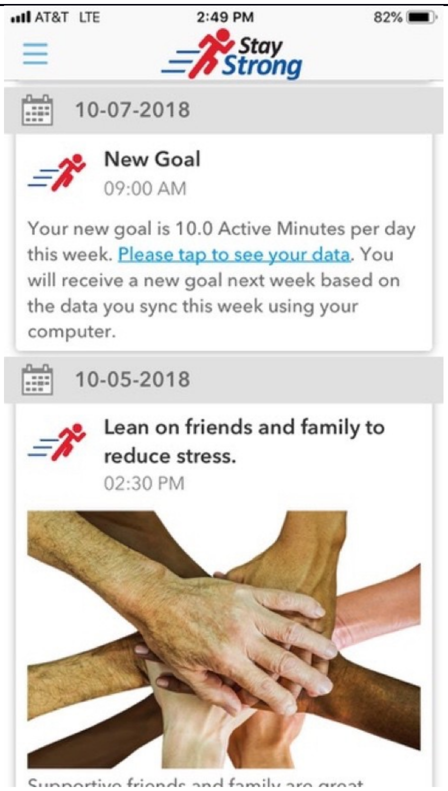

Wall with new goal message and motivational message

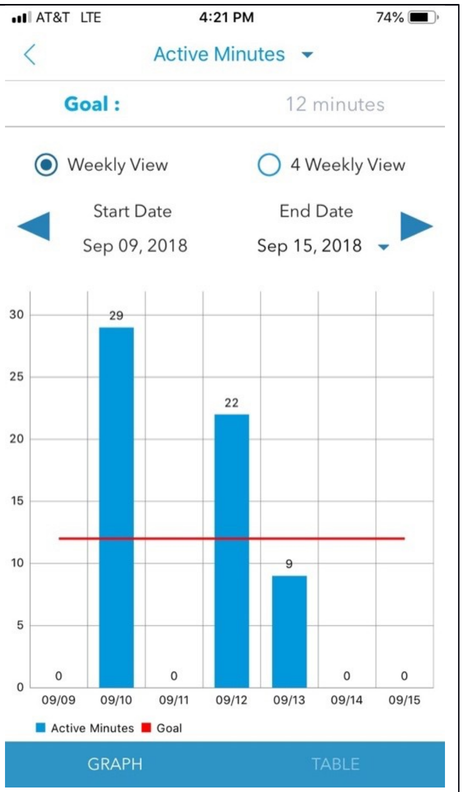

Wall with new goal message and motivational message

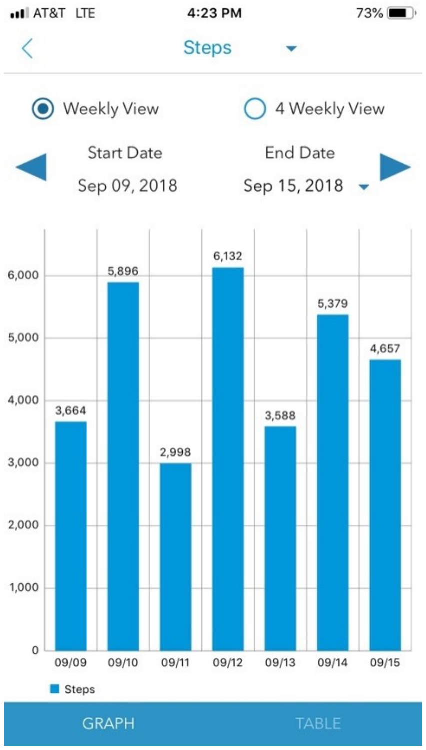

Step counts 1 week graph view

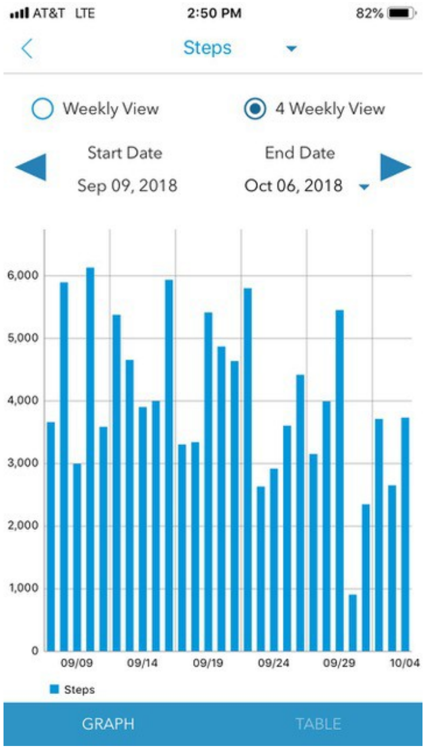

Step counts 4-week graph view

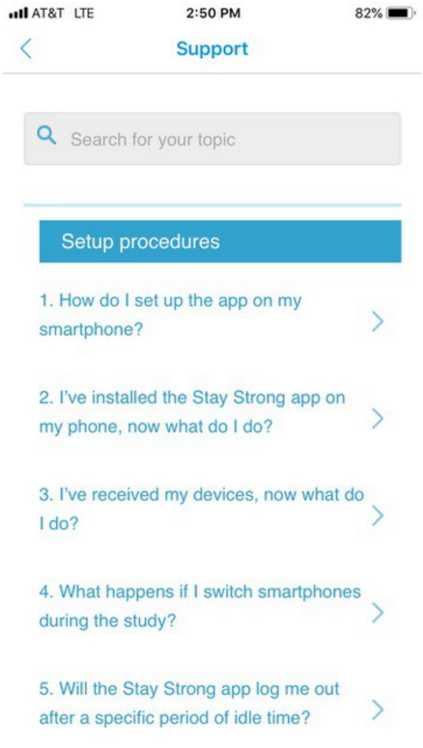

Frequently Asked Questions
