# Supplementary material for: Understanding the Effect of Adding Automated and Human Coaching to a Mobile Health Physical Activity App for Afghanistan and Iraq Veterans: Protocol for a Randomized Controlled Trial of the Stay Strong Intervention
Source: JMIR Res Protoc. 2019 Jan 29;8(1):e12526. doi: 10.2196/12526 (PMC6371069; doi:10.2196/12526)
Supplement: Multimedia Appendix 2 [file resprot_v8i1e12526_app2.pdf]

Multimedia Appendix 2: Behavior Change Techniques (BCTs) by Stay Strong Intervention Component

BCT Technique	Wearable device for passively collected PA (Fitbit Charge 2)	Bluetooth-enabled scale	Behavior data dashboard within Smartphone App	Automated messages*	Telephone-based Lifestyle coaching*
**1.Goals and planning**					
1.1 Goal setting (behavior)			✓	✓	
1.2 Problem solving					✓
1.4 Action planning					✓
1.5. Review behavior goal(s)			✓*		✓
1.6. Discrepancy between current behavior and goal			✓*		✓
1.9. Commitment					✓
**2. Feedback and monitoring**					
2.2 Feedback on behavior	✓	✓	✓		

BCT Technique	Wearable device for passively collected PA (Fitbit Charge 2)	Bluetooth-enabled scale	Behavior data dashboard within Smartphone App	Automated messages*	Telephone-based Lifestyle coaching*
2.3 Self-monitoring of behavior	▯		▯		
2.4 Self-monitoring of behavioral outcomes		▯	▯		
**3. Social support**					
3.1 Social support (unspecified)					▯
**4. Shaping knowledge**					
4.1 Instruction on how to perform a behavior				▯	▯
4.2 Information about antecedents				▯	▯
4.3. Re-attribution					▯
4.4. Behavioral experiments					▯
**5. Natural consequences**					
5.1 Information about health consequences				▯	
5.2 salience of consequences				▯	

BCT Technique	Wearable device for passively collected PA (Fitbit Charge 2)	Bluetooth-enabled scale	Behavior data dashboard within Smartphone App	Automated messages*	Telephone-based Lifestyle coaching*
5.3 Information about social and environmental consequences				✓	
5.6 Information about emotional consequences				✓	
**7. Associations**					
7.1 Prompts/ Cues			✓	✓	
**8. Repetition and substitution**					
8.2 Behavior substitution				✓	
8.3 Habit formation				✓	
8.4 Habit reversal				✓	
8.6 Generalization of a target behavior				✓	
8.7 Graded tasks				✓	
**10. Reward and threat**					
10.9 Self-reward				✓	

BCT Technique	Wearable device for passively collected PA (Fitbit Charge 2)	Bluetooth-enabled scale	Behavior data dashboard within Smartphone App	Automated messages*	Telephone-based Lifestyle coaching*
**11. Regulation**					
11.2 Reduce negative emotions				▯	
11.3 conserving mental resources				▯	
**12. Antecedents**					
12.1 Restructuring the physical environment				▯	
12.2 Restructuring social environment				▯	
12.3 Avoidance/ reducing exposure to cues for the behavior				▯	
12.4 Distraction				▯	
**15. Self-belief**					
15.1 Verbal persuasion about capability				▯	
15.3 Focus on past success				▯	

* *Stay Strong* with Coaching Only
